# Can Tumor Location on Pre-treatment MRI Predict Likelihood of Pseudo-Progression vs. Tumor Recurrence in Glioblastoma?—A Feasibility Study

**DOI:** 10.3389/fncom.2020.563439

**Published:** 2020-12-14

**Authors:** Marwa Ismail, Virginia Hill, Volodymyr Statsevych, Evan Mason, Ramon Correa, Prateek Prasanna, Gagandeep Singh, Kaustav Bera, Rajat Thawani, Manmeet Ahluwalia, Anant Madabhushi, Pallavi Tiwari

**Affiliations:** ^1^Department of Biomedical Engineering, Case Western Reserve University, Cleveland, OH, United States; ^2^Department of Neuroradiology, Imaging Institute, Cleveland Clinic, Cleveland, OH, United States; ^3^Department of Radiology, Northwestern University Feinberg School of Medicine, Chicago, IL, United States; ^4^Department of Biomedical Informatics, Stony Brook University, Stony Brook, NY, United States; ^5^Maimonides Medical Center, New York, NY, United States; ^6^Brain Tumor and Neuro-Oncology Center, Cleveland Clinic, Cleveland, OH, United States; ^7^Louis Stokes Cleveland Veterans Administration Medical Center, Cleveland, OH, United States

**Keywords:** glioblastoma, tumor recurrence, pseudo-progression, atlas, ADIFFI

## Abstract

A significant challenge in Glioblastoma (GBM) management is identifying pseudo-progression (PsP), a benign radiation-induced effect, from tumor recurrence, on routine imaging following conventional treatment. Previous studies have linked tumor lobar presence and laterality to GBM outcomes, suggesting that disease etiology and progression in GBM may be impacted by tumor location. Hence, in this feasibility study, we seek to investigate the following question: Can tumor location on treatment-naïve MRI provide early cues regarding likelihood of a patient developing pseudo-progression vs. tumor recurrence? In this study, 74 pre-treatment Glioblastoma MRI scans with PsP (33) and tumor recurrence (41) were analyzed. First, enhancing lesion on Gd-T_1w_ MRI and peri-lesional hyperintensities on T_2w_/FLAIR were segmented by experts and then registered to a brain atlas. Using patients from the two phenotypes, we construct two atlases by quantifying frequency of occurrence of enhancing lesion and peri-lesion hyperintensities, by averaging voxel intensities across the population. Analysis of differential involvement was then performed to compute voxel-wise significant differences (*p*-value < 0.05) across the atlases. Statistically significant clusters were finally mapped to a structural atlas to provide anatomic localization of their location. Our results demonstrate that patients with tumor recurrence showed prominence of their initial tumor in the parietal lobe, while patients with PsP showed a multi-focal distribution of the initial tumor in the frontal and temporal lobes, insula, and putamen. These preliminary results suggest that lateralization of pre-treatment lesions toward certain anatomical areas of the brain may allow to provide early cues regarding assessing likelihood of occurrence of pseudo-progression from tumor recurrence on MRI scans.

## Introduction

A significant challenge in management of Glioblastoma (GBM), the most aggressive form of brain cancer, is differentiating tumor recurrences from pseudo-progression (PsP) on routine magnetic resonance (MR) scans (Parvez et al., 2014). PsP is a benign radiation-induced treatment effect which occurs in approximately 19–33% of all malignant brain tumors (Wang et al., 2016) and usually stabilizes or regresses without further treatment. Unfortunately, PsP mimics tumor recurrence radiologically on routine MRI scans [Gadolinium-enhanced T1-weighted (Gd-T1w), T2-weighted (T2w), FLAIR], making it challenging to differentiate from true tumor recurrence (Wang et al., 2016). Studies have previously explored advanced imaging modalities such as perfusion imaging (Prager et al., 2015; Chuang et al., 2016; Detsky et al., 2017), MR spectroscopy (Chuang et al., 2016), and diffusion-weighted imaging (Prager et al., 2015) in distinguishing tumor recurrence from PsP. However, these advanced imaging modalities are limited by acquisition variability, costs, reproducibility, and unavailability at most clinical sites (Brandsma et al., 2008). Reliable disease assessment using routine imaging is thus needed in order to aid in accurately identifying PsP from tumor recurrence. Timely identification of these conditions could avoid unnecessary interventions in patients with PsP, while allowing for change in treatment for patients with tumor recurrence (Parvez et al., 2014).

Multiple studies have linked initial lesion location in the brain to be a prognostic marker of tumor recurrence and overall survival in diffuse Gliomas (Ellingson et al., 2012). For instance, recent studies have demonstrated a higher rate of 1p19q deletion in the frontal lobe (Laigle-Donadey et al., 2004), and absence of 1DH1 mutation within the insula (Metellus et al., 2010). Similarly, Gliomas in the frontal locations have been shown to be associated with a better prognosis compared to other locations (Stockhammer et al., 2012). Further, enhancing lesion developing in the periventricular region has been linked to PsP (Patel et al., 2014; Van West et al., 2017). These studies seem to suggest that the underlying disease etiology may be driven by tumor location. Hence, it may be reasonable to rationalize that initial GBM location in the brain may implicitly contribute to an increased likelihood of a patient developing pseudo-progression or tumor recurrence, following conventional treatment of maximal surgical resection and chemo-radiation therapy.

In this feasibility study, we evaluate this hypothesis that lesion location on pre-treatment MR scans could provide early cues regarding likelihood of a patient developing tumor recurrences vs. PsP. In order to anatomically localize the disease, we employ “population atlases” of GBM phenotypes to establish predisposition of tumor recurrence or PsP to specific spatial locations in the brain based on their frequency of occurrence (Larjavaara et al., 2007; Ellingson et al., 2012; Bilello et al., 2016). The statistical population atlases allow for the succinct encapsulation of structural and anatomical variability of the disease across a patient population using a single reference or canonical representation. We will construct population atlases on a cohort of 74 brain MRI scans across two lesion sub-compartments (peritumoral hyperintensities as defined on FLAIR scans and enhancing core as defined on T_1w_ MRI), to quantify the frequency of occurrence of PsP and tumor recurrence in pre-treatment lesions. We will further employ a statistical mapping technique, ADIFFI, to identify if there exist any statistically significant lesion locations in the brain across the two disease pathologies, by comparing the population atlases of PsP and tumor recurrence.

## Materials and Methods

### Study Population

The Institutional Review Board-approved and HIPAA-compliant study comprised GBM patient population from Cleveland Clinic. The population cohort for pre-treatment cases included 74 cases in total; 41 tumor recurrence cases, and 33 PsP cases. All cases were confirmed for disease presence using the criteria provided below. Informed consent was obtained for all patients involved in the study. All MR scans were acquired using either a 1.5 Tesla or a 3-Tesla scanner. Table 1 summarizes the demographics for this study population.

**Table 1:** Summary of the study population used in this work to create population atlases for PsP and tumor recurrence.

**Characteristic**	**Tumor recurrence**	**Pseudo-progression**
No. of patients	41	33
Females	16	12
Males	25	21
Mean age (year)	59.1	61.96
Age range (year)	26–75	24–75

### Confirmation for Disease Presence

Our dataset was identified by performing a retrospective review of all brain tumor patients who had an enhancing lesion within 6 months of treatment (treatment strategies for each patient are provided in Supplementary Material). Our inclusion criteria consisted of the following: (1) pre-, and post-treatment MRI scans that are of diagnostic image quality as determined by collaborating radiologists, (2) availability of all 3 routine MRI sequences (Gd-T1w, T2w, FLAIR), (3) a suspected post-treatment enhancing lesion with more than 5 millimeters (mm) of rim or nodular enhancement, and (4) confirmation of PsP or tumor recurrence for the suspected lesion.

In order to carefully assess the presence of PsP/recurrence, the following steps were followed. First, MRI and other advanced imaging scans (if available) were read by a neuro-radiologist (board-certified in neuroradiology, CAQ) to identify the presence of PsP/recurrence using image assessment based on the RANO criteria (Wen et al., 2010). Then, the initial interpretation was reviewed by patient's clinical team (Neuro-oncology staff, radiation oncologist). All cases were later discussed at a multidisciplinary tumor board in order to provide the final decision. The tumor board constituted of 2+ neuro-oncologists, a neuro-radiologist, a neuropathologist, and one or more surgeons, at our collaborating institution (CCF). A consensus opinion on each individual case was finally formed based on a methodical review of the clinical assessment, prior therapies, and assessment based on imaging features, to identify every study as PsP or tumor recurrence.

### Image Registration and Tumor Segmentation

Manual segmentations in our work were carefully performed by our collaborating experts, where every 2-D slice of each MRI scan (for *n* = 74 studies) with visible tumor was manually annotated [using 3D Slicer (Kikinis et al., 2014)] into 2 regions: enhancing lesion and T_2w_/FLAIR hyperintense peri-lesional component. Gd-T_1w_ MRI scans were used to delineate the enhancing lesion, while both T_2w_ and FLAIR scans were used to annotate the T_2w_/FLAIR hyperintense peri-lesional compartment. A total of four experts were asked to perform the manual annotations. The senior-most expert (V.H expert 1, >10-years of experience in neuroradiology) independently annotated half of the studies, while expert 2 (V.S) with 7 years of experience in neuroradiology supervised expert 3 (K.B, with >3 years of radiology experience, and G. S. with >3 years of experience), to manually annotate the remaining cases individually. In rare cases with disagreement across the readers (expert 2, expert 3, and expert 4), the senior-most radiologist (V.H, expert 1) was consulted to reach consensus and obtain the final segmentations.

In order to map all scans to the same space for the purpose of spatial atlas construction, the Gd-T_1w_ MRI sequence of each patient was co-registered to a healthy 1.0-mm isotropic T1-weighted brain atlas (MNI152; Montreal Neurological Institute), using mutual-information-based similarity measure provided in ANTs (Advanced Normalization Tools) SyN (Symmetric Normalization) toolbox (Avants et al., 2008). This toolbox was employed due to its proved efficiency in mapping brain images containing lesions into healthy templates (Eloyan et al., 2014). In order to ensure exclusion of intensity differences within the tumor regions while only considering intensity differences from healthy tissue, the entire tumor mask was removed during registration. Skull stripping was then performed using a deformable surface classification algorithm (Tao and Chang, 2010), followed by bias field correction that was performed using the non-parametric non-uniform intensity normalization technique in Tustison et al. (2010).

### Frequency Map Construction

From the available annotations for both enhancing lesion and T_2w_/FLAIR hyperintense peri-lesional compartments, population atlases for each compartment were built for both pathologies (tumor recurrence and PsP). These atlases were constructed to quantify the frequency of occurrence of both enhancing lesion and peri-lesional hyperintensities across tumor recurrence and PsP, by averaging intensity values for all voxels across all the annotated binary images of all patients involved in the study. The frequency of lesion occurrence was visualized using a heat map superimposed on the reference MNI152 atlas.

### Analysis of Differential Involvement (ADDIFI)

From the constructed tumor progression and PsP frequency atlases, analysis of differential involvement (ADIFFI) was performed as described in Ellingson et al. (2012), once for the enhancing lesion compartment and once for the peri-lesional hyperintensities. ADIFFI has been previously applied and shown success in the literature in the context of similar clinical problems (Ellingson et al., 2012; Kinoshita et al., 2014). ADIFFI employs Fisher's exact test on a voxel-wise basis, where the test yields exact p-values based on contingency tables (McDonald, 2009). Fisher's exact test is also recommended in the cases with two nominal variables, where there is a need to assess whether the proportions of one variable are different depending on the value of the other variable (McDonald, 2009).

First, a two-tailed Fisher's exact test was conducted, to evaluate a 2 x 2 contingency table that compares tumor recurrence/PsP along with tumor/non-tumor occurrence for each voxel across all patients. From this voxel-wise analysis, significance level was then measured, and the voxels that yielded *p*-value < 0.05 were stored. The voxel-wise probabilities according to Fisher's exact test are computed using the following formula:

$$p=\frac{(a+b)!(c+d)!(a+c)!(b+d)!}{a!b!c!d!n!},$$

where *a, b, c, d*, and *n* are defined as follows:

*a*: represents the number of tumor recurrence as well as the lesion-positive occurrences across all subjects at the current voxel.
*b*: represents the number of tumor progression as well as the lesion-negative occurrences across all subjects at the current voxel.
*c*: represents the number of PsP as well as the lesion-positive occurrences across all subjects at the current voxel.
*d*: represents the number of PsP as well as the lesion-negative occurrences across all subjects at the current voxel.
*n*: represents the total number of studies.

Next, connected component analysis was applied (Vincent, 1993), to cluster all statistically significant voxels found across the two compartments for both tumor recurrence and PsP that appeared on the ADIFFI maps, for enhancing lesion as well as for peri-lesional hyperintensities. The brain was finally partitioned using pre-labeled anatomical structures in MNI space (Mazziotta et al., 2001), for the purpose of identifying the anatomic areas of localization for tumor recurrence/PsP across all subjects.

### Cluster-Size Correction Using Random Permutation Analysis

Due to the extensive number of voxel-wise calculations performed during ADIFFI, multiple comparison corrections were performed, which also aim at isolating the spatially distinct clusters associated with significant differences between the two groups. Random permutation (RP) analysis was conducted for cluster size correction (Bullmore et al., 1999). Specifically, our task was to determine to what extent we can randomly obtain a cluster of statistical significance the same size or larger than the observed pattern in the original tumor recurrence versus PsP statistical maps. In order to achieve this, all T_2w_/FLAIR hyperintense peri-lesional components, as well as the enhancing lesion ones, across the two categories (tumor recurrence/ PsP) were randomly reassigned to one of these pathologies, then ADIFFI was re-conducted, and voxels with *p*-values < 0.05 were stored. In addition, the sizes of statistically significant clusters were documented at each iteration. The whole process was reiterated across 500 iterations. RP analysis was employed in order to identify distinct clusters occurring <5% by chance, which would provide distinct spatial differences between tumor recurrence and PsP.

**Figure 1:**
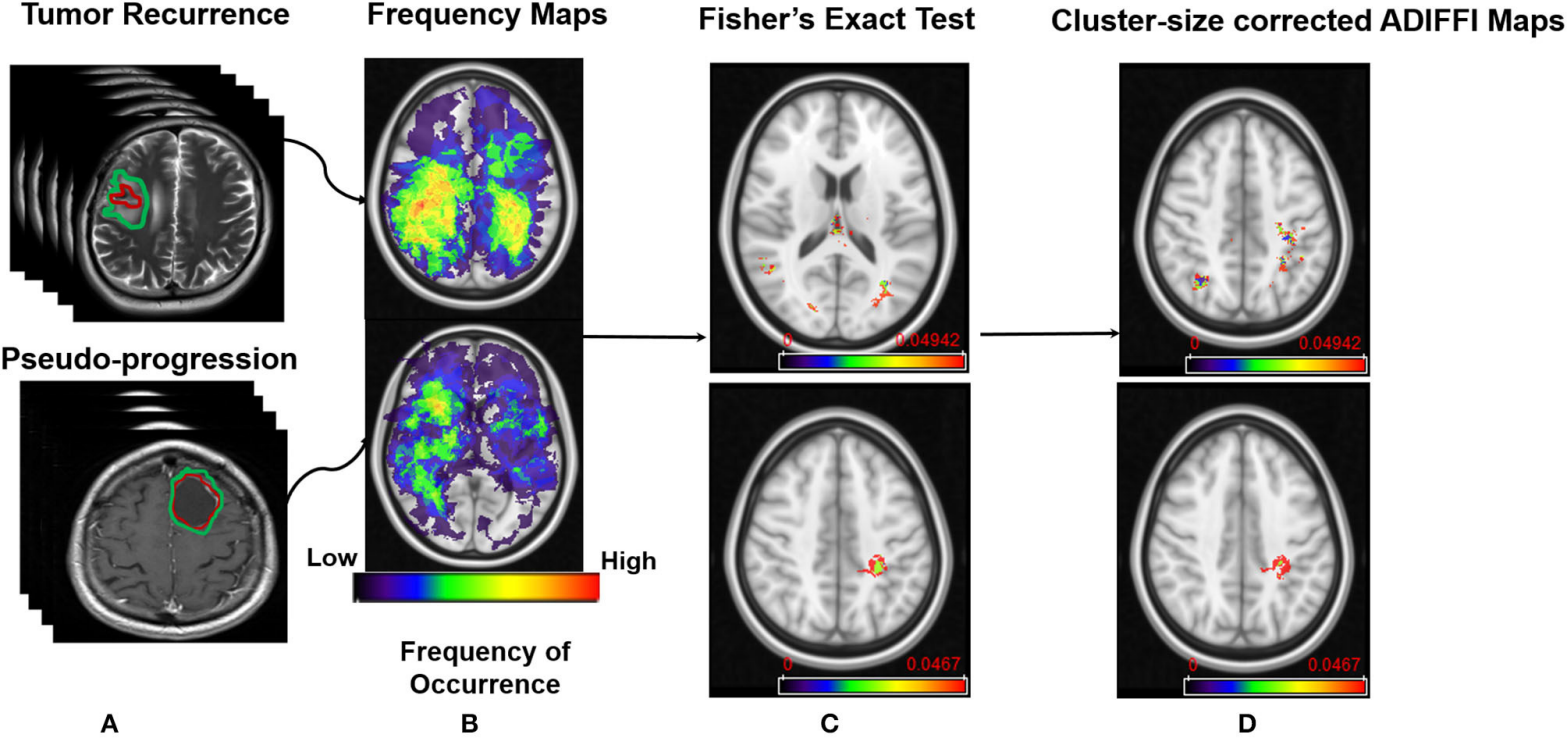
Pipeline of the framework. **(A)** MR scans of tumor recurrence and pseudo-progression. **(B)** Frequency map atlases that were constructed from the two classes. **(C)** Results from Fisher's Exact test on peri-lesional T2/FLAIR hyperintensities in tumor recurrence (Top), and enhancing lesion in tumor recurrence (Bottom). **(D)** Results after applying RP analysis on ADIFFI maps shown in **(C)**.

Finally, statistically significant clusters appearing on the cluster-size corrected ADIFFI maps were designated as either PsP or tumor recurrence by referring to the population atlases that were individually constructed for tumor recurrence and PsP. A specific anatomic localization was then obtained from these cluster-size corrected ADIFFI maps, by mapping them to a structural MNI atlas. The entire pipeline of this work is shown in Figure 1.

## Results

**Figure 2:**
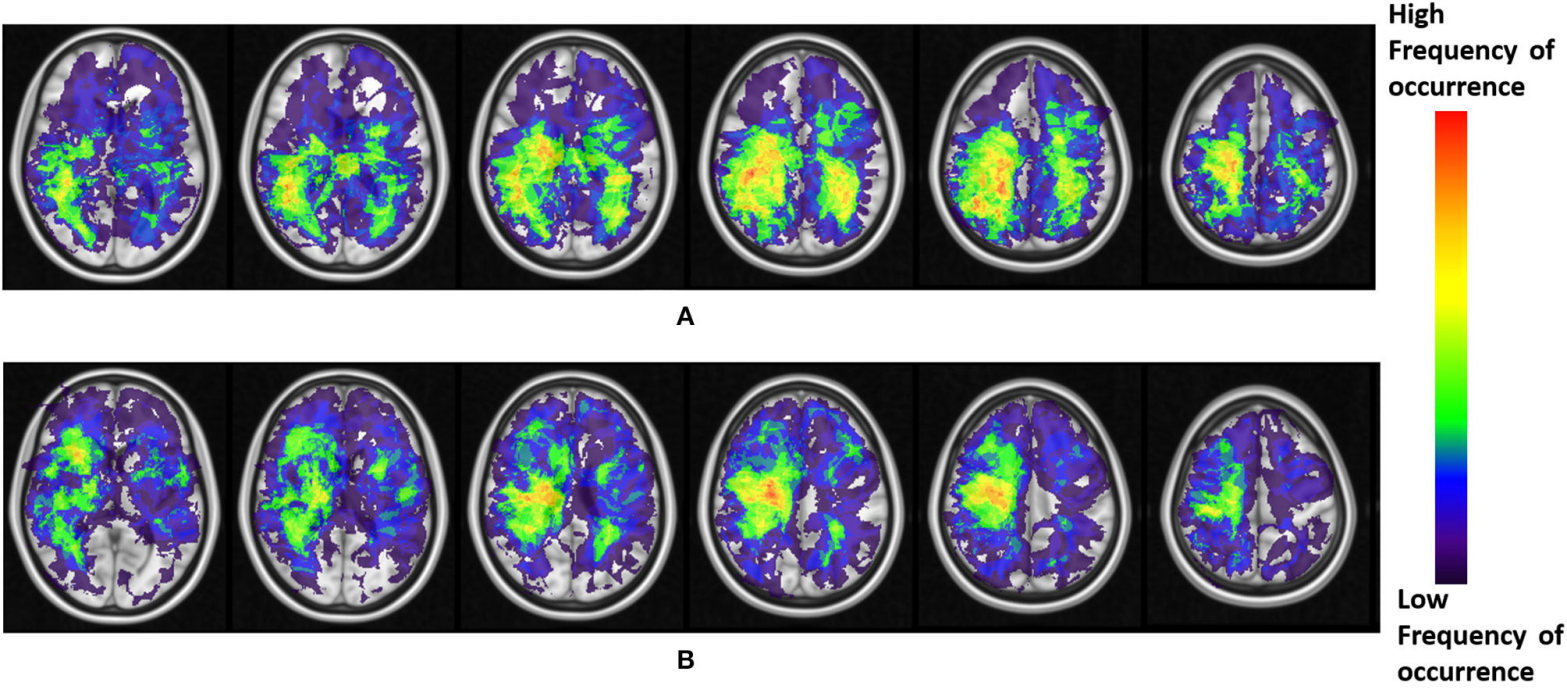
**(A)** Frequency maps of tumor occurrence for peri-lesional T2/FLAIR hyperintensities in tumor recurrence of pre-treatment scans, where lobar prominence is present in the parietal lobe of both hemispheres. **(B)** Frequency maps of tumor occurrence for peri-lesional T2/FLAIR hyperintensities in pseudo-progression, where a multi-focal distribution is present in the frontal lobe, temporal lobe, insula, and putamen of the right hemisphere.

**Figure 3:**
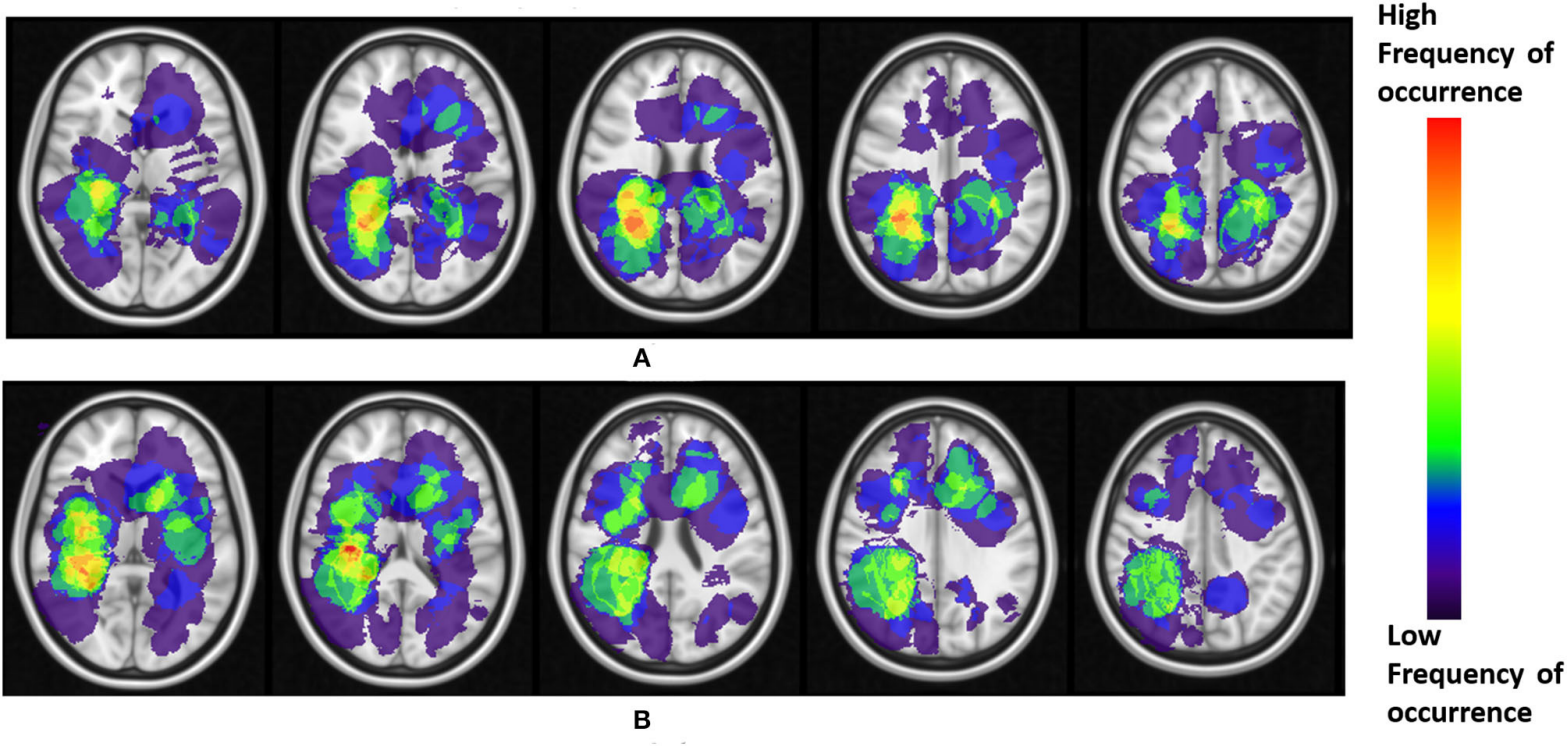
**(A)** Frequency maps of tumor occurrence for enhancing lesion in tumor recurrence of pre-treatment scans, where lobar prominence is present in the parietal lobe of both hemispheres. **(B)** Frequency maps of tumor occurrence for enhancing lesion in pseudo-progression, where a multi-focal distribution is present in the insula, frontal lobe, putamen, and the temporal lobe, of both left and right hemispheres.

The resulting frequency maps that were constructed for both T2w/FLAIR hyperintense peri-lesional and lesion areas from pre-treatment scans are shown in Figures 2, 3, respectively. These figures show that tumor recurrence in both compartments (enhancing lesion and T2w/FLAIR hyperintense peri-lesional areas) is more likely lateralized toward the parietal lobe, whereas PsP is more likely to be multi-focally distributed across different anatomical areas of the brain including frontal and temporal lobes, the insula, and the putamen.

### Tumor Recurrence Is Lateralized Toward the Parietal Lobe

**Figure 4:**
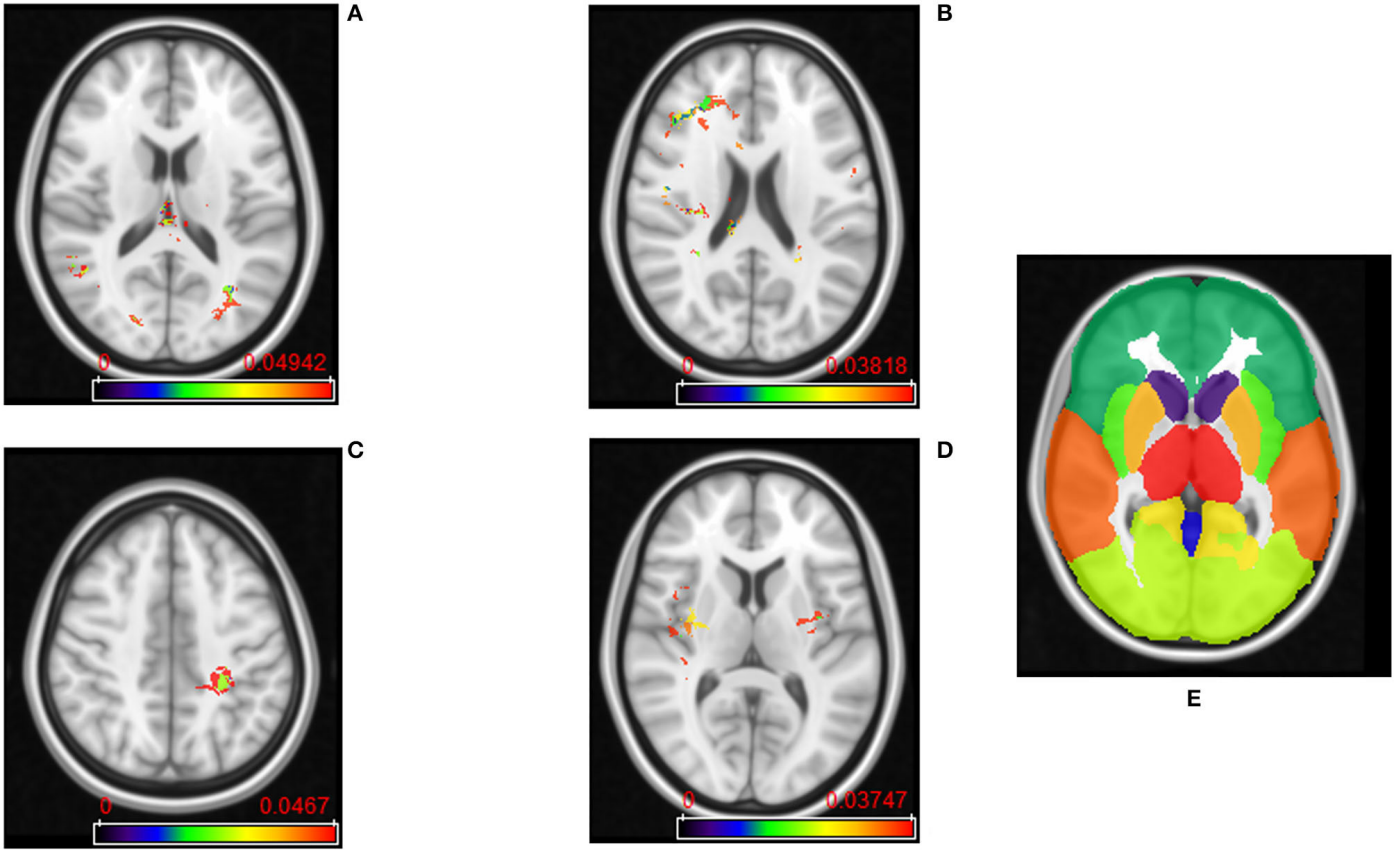
**(A)** ADIFFI maps for peri-lesional T2/FLAIR hyperintensities in tumor recurrence, and **(B)** pseudo-progression. **(C)** ADIFFI maps for enhancing lesion in tumor recurrence, and **(D)** pseudo-progression. The level of significance was at a *p*-value of 0.05 for all of these maps. These were the maps prior to applying RP analysis. **(E)** The labeled anatomical MNI atlas that is used for parcellating ADIFFI maps and identifying significant areas.

The frequency maps as well as ADIFFI maps for peri-lesional T2/FLAIR hyperintensities of the pre-treatment scans show that tumor recurrence is more likely to be present in the parietal lobe, with frequency of occurrence of 85% (59% of this distribution was found in the right hemisphere, whereas 41% was found in the left hemisphere), 13% in the occipital lobe (83% in the right hemisphere and 17% in the left hemisphere), and 2% in the right temporal lobe (Figures 2A, 4A). Frequency maps as well as ADIFFI maps obtained for the enhancing lesion also reveal that tumor recurrence is more likely to be present in the parietal lobe of left and right hemispheres (70% and 30% chances of occurrence, respectively), Figures 3A, 4C. These results suggest that tumor recurrence exhibits lobar prominence across the population atlases, but do not exhibit any hemisphere-specific preference. These lobar percentages were obtained by parcellating the brain with respect to an MNI structural atlas, shown in Figure 4E.

### Pseudo-Progression Exhibits a Multi-Focal Distribution in the Enhancing Lesion as Well as the Perilesional Hyperintensities

PsP, unlike tumor recurrence, seems to more likely be multi-focally distributed across the brain in pre-treatment cases, for both the enhancing lesion and the peri-lesional hyperintensities. PsP exhibited a multi-focal distribution in the right hemisphere of the peri-lesional hyperintensities, with frequencies of occurrence of 55% in the frontal lobe, 11% in the temporal lobe, 10% in the insula, 10% in the putamen, and 9% in the parietal lobe (77% in the right hemisphere and 23% in the left hemisphere), and 5% in the right thalamus (Figures 2B, 4B). In the analysis of the enhancing lesion regions, PsP appears to more likely be multi-focally distributed within both left and right hemispheres. The spatial distribution was 35% in the insula (with 63% of this distribution in the right hemisphere and 37% in the left hemisphere), 21% in the right frontal lobe, 13% in the right temporal lobe, 17% in the putamen (with 57% of this distribution in the right hemisphere and 43% in the left hemisphere), and 14% in the right parietal lobe (Figures 3B, 4D).

### Random Permutation Analysis for Cluster Size Correction

RP analysis conducted on the peri-lesional T2/FLAIR hyperintensities of the pre-treatment cases revealed that the average and standard deviation of maximum cluster size are 3,700 and 1726.8 voxels, respectively. Also, 95% of the cluster sizes were smaller than 6,192 voxels, meaning that clusters larger than this size threshold would occur in <5% of all random permutations. This resulted in one distinct T2w/FLAIR hyperintense peri-lesional cluster size of 6,502 voxels, localized at the right parietal lobe, and associated with tumor recurrence, and another one of size of 6,200 voxels localized at the left parietal lobe.

RP analysis conducted on the enhancing lesion revealed that average and standard deviation of maximum cluster size are 2,258 and 1774.1 voxels, respectively. Also, 95% of the cluster sizes were smaller than 5,164 voxels, meaning that clusters larger than this size threshold would occur in <5% of all random permutations. This resulted in one distinct enhancing lesion cluster size of 5,450 voxels, localized at the left parietal lobe, and associated with tumor recurrence.

The designation of PsP or true progression based on ADIFFI maps as for each significant voxel/cluster was accomplished by referring to the population atlases of both compartments (enhancing lesion, T2w/FLAIR hyperintense peri-lesion) that were individually constructed for tumor recurrence and PsP. The cluster-size corrected ADIFFI maps obtained for tumor recurrence are shown in Figure 1D.

Apart from the probabilistic approach conducted above, we conducted a statistical experiment, where we used a two-sample *t*-test, that was performed on the clinical parameters of tumor recurrence versus pseudo-progression, namely extent of resection, age, and gender, to obtain the 5% significance level. We found that the difference between the two pathologies was not statistically significant, based on each of the 3 parameters, *p*-values = 0.52, 0.25, and 0.82 for extent of resection, age, and gender, respectively. All of this information is available in Supplementary Material.

## Discussion

Distinguishing tumor recurrence from PsP is one of the biggest clinical challenges in GBM management. This feasibility study aimed at creating population atlases to study spatial proclivity of brain tumor recurrence vs. PsP based on their occurrences on pre-treatment MR scans. The study assessed the voxel-wise tumor frequency across two lesion compartments using a statistical mapping technique named ADIFFI, in efforts to find significant spatial distribution differences between the two phenotypes.

Our preliminary findings suggest that likelihood of tumor recurrence is more consistent with lesions occurring in the parietal lobe of both left and right hemispheres, based on the analysis of both enhancing lesion and peri-lesional T2/FLAIR hyperintensities, on pre-treatment MRI scans. Parietal lobe is largely responsible for cognitive functions. Damage to parietal lobe may have direct implications in processing speech as well as sensory information. Hence, presence of tumor recurrence in parietal lobe may cause symptoms associated with numbness and tingling, hemi-neglect, and cognitive issues around right-left confusion and reading and math problems. PsP, on the other hand, did not exhibit lobar-specific distribution in pre-treatment scans, but showed a multi-focal distribution of the initial tumor in the frontal (associated with motor function, memory, problem solving) and temporal lobes (associated with primary auditory perception, such as hearing and visual recognition) as well as the insula and putamen. While the association of presence of tumor recurrence or PsP with specific lobes in the brain is not well-understood, their presence in specific lobes could ultimately contribute toward making more informed decisions regarding their diagnosis.

Previous studies have largely employed population atlases in brain tumors using pre-treatment MRI to obtain probabilistic maps of spatial predisposition in patients based on their disease aggressiveness (Duffau and Capelle, 2004) or molecular status (Drabycz et al., 2010; Ellingson et al., 2012; Kanas et al., 2017). For instance, a few studies have shown that tumor recurrence closer to the ventricular system was significantly associated with poor survival (Jafri et al., 2012; Adeberg et al., 2014). Interestingly, the study in Liu et al. (2016) showed that tumors in the right occipito-temporal periventricular white matter were significantly associated with poor survival in both training and test cohorts. Similarly, more aggressive GBMs were reported to be close to the ventricular system, and had a rapid progression (Li et al., 2018), suggesting that tumor location may play a significant role in disease etiology.

The closest studies to our work have attempted to identify associations of lesion location with likelihood of tumor recurrence and PsP, to investigate any spatial differences between the two phenotypes. For instance, the study by Tsien et al. (2010) incorporated location along with clinical and conventional MRI parameters to distinguish tumor progression from PsP in high-grade gliomas, yet no significant location differences could be found between the two groups, perhaps on account of the relatively small population size involved in this study (27 patients total). The study by Van West et al. (2017) reported the incidence of PsP in low grade gliomas, and found that 50% of their PsP enhancing lesions were located in the periventricular walls; attributing to the relatively poor blood supply in the periventricular areas that make it more vulnerable to radiation-induced processes. However, these studies did not report any findings regarding lobular preferences for either PsP or tumor recurrence in GBMs.

Our study did have its limitations. First, our dataset is relatively small (74 studies). However, our sample size of *n* = 74 studies is comparable to existing studies in the literature on distinguishing PsP from tumor recurrence with sample sizes ranging from *n* = 19 to *n* = 98 (Cha et al., 2014; Wang et al., 2016; Boxerman et al., 2017; Elshafeey et al., 2019). Additionally, our work, similar to some of the published studies in distinguishing PsP vs. tumor recurrence (Cha et al., 2014; Elshafeey et al., 2019), did not include a separate hold-out validation cohort for analysis. Future work will focus on obtaining additional pre-treatment cases to further investigate our spatial predisposition findings, for tumor recurrence and pseudo-progression on large multi-institutional studies, as well as validate our findings on a separate independent patient cohort. In addition, while our results are promising as a feasibility study, our study did not account for molecular status (i.e., MGMT), or Karnofsky performance score as potential confounders during analysis. A potential limitation of this study is the lack of advanced imaging modalities such as dynamic susceptibility contrast (DSC), and Fluoro-O-(2) fluoroethyl-l-tyrosine (FET), which could have allowed for a joint multi-modal analysis combining these modalities with the probabilistic atlases. Additionally, one of our future directions includes extensively evaluating different automated segmentation approaches on the tumor compartments for the constructed probabilistic atlases, to extend our feasibility analysis. We also plan to obtain multiple segmentations from different readers for every study, to assess the impact of segmentation variability on our analysis. The prognostic implications (i.e., predicting patient overall survival), based on the location differences across PsP and tumor recurrence will also be investigated in the future.

To conclude, this study attempted to demonstrate the likelihood of occurrence of tumor recurrence and pseudo-progression, using the location of the lesion on pre-treatment MR scans. Our results revealed distinct localization between tumor recurrences and PsP that could aid in predicting these two similar appearing pathological conditions. Future work will focus on integrating the location biomarker with other biomarkers, such as shape and texture features, on a larger cohort of multi-institutional studies. We will also consider identifying location specific markers associated with radiation necrosis (delayed treatment effects) vs. tumor recurrence.

## Data Availability Statement

The raw data supporting the conclusions of this article will be made available by the authors, without undue reservation.

## Ethics Statement

The studies involving human participants were reviewed and approved by Cleveland Clinic Institutional Review Board. The patients/participants provided their written informed consent to participate in this study.

## Author Contributions

MI and PT contributed to analysis and interpretation of data and contributed to designing the experiments, drafting, and revising the article. VH, VS, and EM provided clinical datasets and interpretation of radiographic images. MA helped define the clinical problem and provided clinical interpretation of findings. VH, RC, PP, GS, KB, and RT curated the studies and performed the annotations on radiological images. AM revised the manuscript critically for important intellectual content. All authors have reviewed the manuscript.

## Conflict of Interest

The authors declare that the research was conducted in the absence of any commercial or financial relationships that could be construed as a potential conflict of interest.

## References

[B1] Adeberg S. König L. Bostel T. Harrabi S. Welzel T. Debus J. (2014). Glioblastoma recurrence patterns after radiation therapy with regard to the subventricular zone. Int. J. Radiat. Oncol. Biol. Phys. 90, 886–893. 10.1016/j.ijrobp.2014.07.027 25220720

[B2] Avants B. B. Epstein C. L. Grossman M. Gee J. C. (2008). Symmetric diffeomorphic image registration with cross-correlation: evaluating automated labeling of elderly and neurodegenerative brain. Med. Image Anal. 12, 26–41. 10.1016/j.media.2007.06.004 17659998 PMC2276735

[B3] Bilello M. Akbari H. Da X. Pisapia J. M. Mohan S. Wolf R. L.. (2016). Population-based MRI atlases of spatial distribution are specific to patient and tumor characteristics in glioblastoma. NeuroImage 12, 34–40. 10.1016/j.nicl.2016.03.007 27358767 PMC4916067

[B4] Boxerman J. L. Ellingson B. M. Jeyapalan S. Elinzano H. Harris R. J. Rogg J. M.. (2017). Longitudinal DSC-MRI for distinguishing tumor recurrence from pseudoprogression in patients with a high-grade glioma. Am. J. Clin. Oncol. 40, 228–234. 10.1097/COC.0000000000000156 25436828

[B5] Brandsma D. Stalpers L. Taal W. Sminia P. van den Bent M. J. (2008). Clinical features, mechanisms, and management of pseudo-progression in malignant gliomas. Lancet Oncol. 9, 453–461. 10.1016/S1470-2045(08)70125-6 18452856

[B6] Bullmore E. T. Suckling J. Overmeyer S. Rabe-Hesketh S. Taylor E. Brammer M. J. (1999). Global, voxel, and cluster tests, by theory and permutation, for a difference between two groups of structural MR images of the brain. IEEE Trans. Med. Imaging 18, 32–42. 10.1109/42.750253 10193695

[B7] Cha J. Kim S. T. Kim H. J. Kim B. J. Kim Y. K. Lee J. Y.. (2014). Differentiation of tumor progression from pseudoprogression in patients with posttreatment glioblastoma using multiparametric histogram analysis. Am. J. Neuroradiol. 35, 1309–1317. 10.3174/ajnr.A3876 24676005 PMC7966603

[B8] Chuang M. T. Liu Y. S. Tsai Y. S. Chen Y. C. Wang C. K. (2016). Differentiating radiation-induced necrosis from recurrent brain tumor using MR perfusion and spectroscopy: a meta-analysis. PloS ONE 11:e0141438. 10.1371/journal.pone.0141438 26741961 PMC4712150

[B9] Detsky J. S. Keith J. Conklin J. Symons S. Myrehaug S. Sahgal A.. (2017). Differentiating radiation necrosis from tumor progression in brain metastases treated with stereotactic radiotherapy: utility of intravoxel incoherent motion perfusion MRI and correlation with histopathology. J. Neurooncol. 134, 433–441. 10.1007/s11060-017-2545-2 28674974

[B10] Drabycz S. Roldán G. De Robles P. Adler D. McIntyre J. B. Magliocco A. M.. (2010). An analysis of image texture, tumor location, and MGMT promoter methylation in glioblastoma using magnetic resonance imaging. Neuroimage 49, 1398–405. 10.1016/j.neuroimage.2009.09.049 19796694

[B11] Duffau H. Capelle L. (2004). Preferential brain locations of low-grade gliomas: comparison with glioblastomas and review of hypothesis. Cancer 100, 2622–2626. 10.1002/cncr.20297 15197805

[B12] Ellingson B. M. Cloughesy T. F. Pope W. B. Zaw T. M. Phillips H. Lalezari S.. (2012). Anatomic localization of O6-methylguanine DNA methyltransferase (MGMT) promoter methylated and unmethylated tumors: a radiographic study in 358 *de novo* human glioblastomas. Neuroimage 59, 908–916. 10.1016/j.neuroimage.2011.09.076 22001163

[B13] Eloyan A. Shou H. Shinohara R. T. Sweeney E. M. Nebel M. B. Cuzzocreo J. L.. (2014). Health effects of lesion localization in multiple sclerosis: spatial registration and confounding adjustment. PloS ONE 9:e107263. 10.1371/journal.pone.0107263 25233361 PMC4169434

[B14] Elshafeey N. Kotrotsou A. Hassan A. Elshafei N. Hassan I. Ahmed S. (2019). Multicenter study demonstrates radiomic features derived from magnetic resonance perfusion images identify pseudo-progression in glioblastoma. Nat. Commun. 10, 1–9. 10.1038/s41467-019-11007-0 31320621 PMC6639324

[B15] Jafri N. F. Clarke J. L. Weinberg V. Barani I. J. Cha S. (2012). Relationship of glioblastoma multiforme to the subventricular zone is associated with survival. Neuro-oncology 15, 91–96. 10.1093/neuonc/nos268 23095230 PMC3534420

[B16] Kanas V. G. Zacharaki E. I. Thomas G. A. Zinn P. O. Megalooikonomou V. Colen R. R. (2017). Learning MRI-based classification models for MGMT methylation status prediction in glioblastoma. Comput. Methods Programs Biomed. 140, 249–257. 10.1016/j.cmpb.2016.12.018 28254081

[B17] Kikinis R. Pieper S. D. Vosburgh K. G. (2014). 3D slicer: a platform for subject-specific image analysis, visualization, and clinical support, in Intraoperative Imaging and Image-Guided Therapy, eds Jolesz F. (New York, NY: Springer), 277–289. 10.1007/978-1-4614-7657-3_19

[B18] Kinoshita M. Sasayama T. Narita Y. Yamashita F. Kawaguchi A. Chiba Y.. (2014). Different spatial distribution between germinal center B and non-germinal center B primary central nervous system lymphoma revealed by magnetic resonance group analysis. Neuro-oncology 16, 728–734. 10.1093/neuonc/not319 24497406 PMC3984556

[B19] Laigle-Donadey F. Martin-Duverneuil N. Lejeune J. Criniere E. Capelle L. Duffau H.. (2004). Correlations between molecular profile and radiologic pattern in oligodendroglial tumors. Neurology 63, 2360–2362. 10.1212/01.WNL.0000148642.26985.68 15623700

[B20] Larjavaara S. Mäntylä R. Salminen T. Haapasalo H. Raitanen J. Jääskeläinen J.. (2007). Incidence of gliomas by anatomic location. Neuro-oncology 9, 319–325. 10.1215/15228517-2007-016 17522333 PMC1907421

[B21] Li H. Y. Sun C. R. He M. Yin L. C. Du H. G. Zhang J. M. (2018). Correlation between tumor location and clinical properties of glioblastomas in frontal and temporal lobes. World Neurosurg. 112, 407–414. 10.1016/j.wneu.2018.01.055 29355809

[B22] Liu T. T. Achrol A. S. Mitchell L. A. Du W. A. Loya J. J. Rodriguez S. A.. (2016). Computational identification of tumor anatomic location associated with survival in 2 large cohorts of human primary glioblastomas. Am. J. Neuroradiol. 37, 621–28. 10.3174/ajnr.A4631 26744442 PMC4833648

[B23] Mazziotta J. Toga A. Evans A. Fox P. Lancaster J. Zilles K.. (2001). A probabilistic atlas and reference system for the human brain: International Consortium for Brain Mapping (ICBM). Philos. Trans. R. Soc. Lond., B, Biol. Sci. 356, 1293–1322. 10.1098/rstb.2001.0915 11545704 PMC1088516

[B24] McDonald J. H. (2009). Handbook of Biological Statistics. Baltimore, MD: Sparky House Publishing

[B25] Metellus P. Coulibaly B. Colin C. de Paula A. M. Vasiljevic A. Taieb D.. (2010). Absence of IDH mutation identifies a novel radiologic and molecular subtype of WHO grade II gliomas with dismal prognosis. Acta Neuropathol. 120, 719–729. 10.1007/s00401-010-0777-8 21080178

[B26] Parvez K. Parvez A. Zadeh G. (2014). The diagnosis and treatment of pseudo-progression, radiation necrosis and brain tumor recurrence. Int. J. Mol. Sci. 15, 11832–11846. 10.3390/ijms150711832 24995696 PMC4139817

[B27] Patel U. Patel A. Cobb C. Benkers T. Vermeulen S. (2014). The management of brain necrosis as a result of SRS treatment for intra-cranial tumors. Transl. Cancer Res. 3, 373–382. 10.3978/j.issn.2218-676X.2014.07.05

[B28] Prager A. J. Martinez N. Beal K. Omuro A. Zhang Z. Young R. J. (2015). Diffusion and perfusion MRI to differentiate treatment-related changes including pseudo-progression from recurrent tumors in high-grade gliomas with histopathologic evidence. Am. J. Neuroradiol. 36, 877–885. 10.3174/ajnr.A4218 25593202 PMC4731220

[B29] Stockhammer F. Misch M. Helms H. J. Lengler U. Prall F. Von Deimling A.. (2012). IDH1/2 mutations in WHO grade II astrocytomas associated with localization and seizure as the initial symptom. Seizure 21, 194–197. 10.1016/j.seizure.2011.12.007 22217666

[B30] Tao X. Chang M. C. (2010). A skull stripping method using deformable surface and tissue classification. Proc. SPIE 7623:76233L 10.1117/12.844061

[B31] Tsien C. Galbán C. J. Chenevert T. L. Johnson T. D. Hamstra D. A. Sundgren P. C. (2010). Parametric response map as an imaging biomarker to distinguish progression from pseudo-progression in high-grade glioma. J. Clin. Oncol. 28:2293 10.1200/JCO.2009.25.3971 20368564 PMC2860441

[B32] Tustison N. J. Avants B. B. Cook P. A. Zheng Y. Egan A. Yushkevich P. A. (2010). N4ITK: improved N3 bias correction. IEEE Trans. Med. Imaging 29, 1310–1320. 10.1109/TMI.2010.2046908 20378467 PMC3071855

[B33] Van West S. E. de Bruin H. G. van de Langerijt B. Swaak-Kragten A. T. Van Den Bent M. J. Taal W. (2017). Incidence of pseudo-progression in low-grade gliomas treated with radiotherapy. Neuro-oncology 19, 719–725. 10.1093/neuonc/now194 28453748 PMC5464441

[B34] Vincent L. (1993). Morphological grayscale reconstruction in image analysis: applications and efficient algorithms. IEEE Trans. Image Process. 2, 176–201. 10.1109/83.217222 18296207

[B35] Wang S. Martinez-Lage M. Sakai Y. Chawla S. Kim S. G. Alonso-Basanta M.. (2016). Differentiating tumor progression from pseudoprogression in patients with glioblastomas using diffusion tensor imaging and dynamic susceptibility contrast MRI. Am. J. Neuroradiol. 37, 28–36. 10.3174/ajnr.A4474 26450533 PMC7960225

[B36] Wen P. Y. Macdonald D. R. Reardon D. A. Cloughesy T. F. Gregory Sorensen A. DeGroot E. G. (2010) Updated response assessment criteria for high-grade gliomas: response assessment in neuro-oncology working group. J. Clin. Oncol. 28, 1963–1972. 10.1200/JCO.2009.26.3541. 20231676

